# Supplementary material for: Nitrogen metabolism profiling reveals cell state-specific pyrimidine synthesis pathway choice
Source: Nat Metab. 2026 Apr 29;8(5):1124–48. doi: 10.1038/s42255-026-01520-0 (PMC13218935; doi:10.1038/s42255-026-01520-0)
Supplement: Supplementary file 1 — Supplementary Fig. 1 [file 42255_2026_1520_MOESM1_ESM.pdf]

nature metabolism

Supplementary information https://doi.org/10.1038/s42255-026-01520-0

Nitrogen metabolism profiling reveals cell state-specific pyrimidine synthesis pathway choice

In the format provided by the authors and unedited

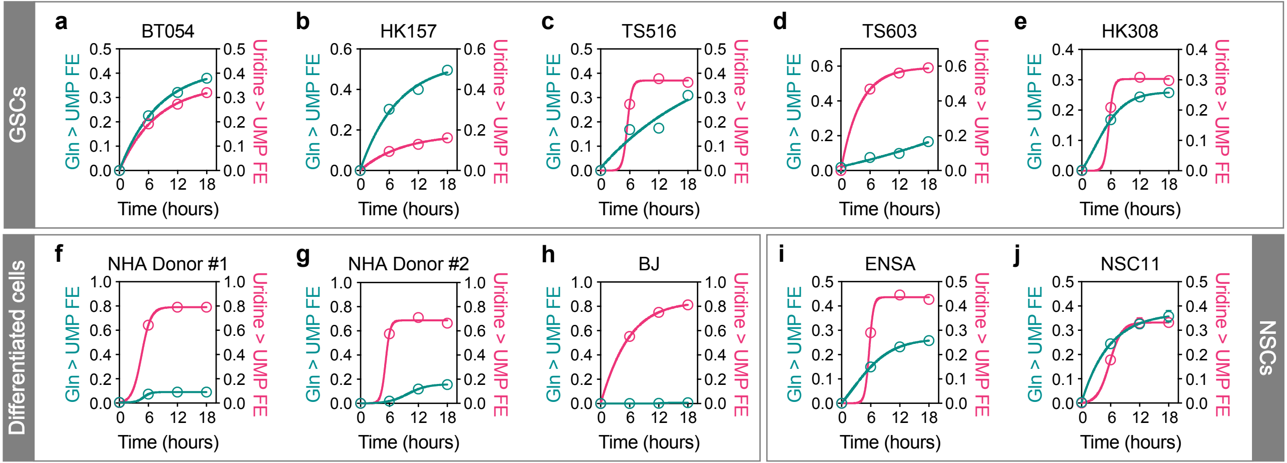

Supplementary Fig. 1. Un-normalized tracing of pyrimidine synthesis in GSCs, differentiated cells, and NSCs, related to Fig. 2. a-j, Amide-^{15}N-glutamine (Gln) and $^{15}N_2$-uridine tracing to UMP in (**a-e**) GSC, (**f-h**) differentiated, or (**i-j**) NSC lines (n = 3 for all except HK308, for which n = 2). In contrast to Fig. 2, amide-^{15}N-glutamine to UMP labeling data are not normalized to glutamine M+1 FE. FE = fractional enrichment. For all panels, data are means ± s.e.m.
