# Supplementary material for: Nitrogen metabolism profiling reveals cell state-specific pyrimidine synthesis pathway choice
Source: Nat Metab. 2026 Apr 29;8(5):1124–48. doi: 10.1038/s42255-026-01520-0 (PMC13218935; doi:10.1038/s42255-026-01520-0)
Supplement: Supplementary file 2 — Reporting Summary [file 42255_2026_1520_MOESM2_ESM.pdf]

nature portfolio

Corresponding author(s): Samuel K. McBrayer

Last updated by author(s): Feb 22, 2026

Reporting Summary

Nature Portfolio wishes to improve the reproducibility of the work that we publish. This form provides structure for consistency and transparency in reporting. For further information on Nature Portfolio policies, see our Editorial Policies and the Editorial Policy Checklist.

Statistics

For all statistical analyses, confirm that the following items are present in the figure legend, table legend, main text, or Methods section.

n/a	Confirmed	
☐	☒	The exact sample size (*n*) for each experimental group/condition, given as a discrete number and unit of measurement
☐	☒	A statement on whether measurements were taken from distinct samples or whether the same sample was measured repeatedly
☐	☒	The statistical test(s) used AND whether they are one- or two-sided<br>*Only common tests should be described solely by name; describe more complex techniques in the Methods section.*
☒	☐	A description of all covariates tested
☐	☒	A description of any assumptions or corrections, such as tests of normality and adjustment for multiple comparisons
☐	☒	A full description of the statistical parameters including central tendency (e.g. means) or other basic estimates (e.g. regression coefficient) AND variation (e.g. standard deviation) or associated estimates of uncertainty (e.g. confidence intervals)
☐	☒	For null hypothesis testing, the test statistic (e.g. *F*, *t*, *r*) with confidence intervals, effect sizes, degrees of freedom and *P* value noted<br>*Give P values as exact values whenever suitable.*
☒	☐	For Bayesian analysis, information on the choice of priors and Markov chain Monte Carlo settings
☒	☐	For hierarchical and complex designs, identification of the appropriate level for tests and full reporting of outcomes
☒	☐	Estimates of effect sizes (e.g. Cohen's *d*, Pearson's *r*), indicating how they were calculated

Our web collection on statistics for biologists contains articles on many of the points above.

Software and code

Policy information about availability of computer code

Data collection	TraceFinder (5.1 SP2), SCiLS Lab (2023a Core), Brainlab Automatic Image Registration
Data analysis	El-MAVEN (0.12.0), TraceFinder (5.1 SP2), R (4.1.1 or 4.4.2), RStudio (2024.09), AccuCor (0.3.0), SCiLS Lab (2023a Core), rMSIproc (0.3.166), enviPat (2.767), GraphPad Prism (10.4.1), PyMol (3.1.1), Clustal Omega (1.2.4), SnapGene (8.1.1), WebLogo 3 (3.9.0), ggalluvial (0.12.5), ggplot2 (3.4.2), FragPipe, Proteome Discoverer (3.0), Fiji/ImageJ2 (1.54p), ELDA (Last Modified October 24, 2014)

For manuscripts utilizing custom algorithms or software that are central to the research but not yet described in published literature, software must be made available to editors and reviewers. We strongly encourage code deposition in a community repository (e.g. GitHub). See the Nature Portfolio guidelines for submitting code & software for further information.

Data

Policy information about availability of data

All manuscripts must include a data availability statement. This statement should provide the following information, where applicable:

- Accession codes, unique identifiers, or web links for publicly available datasets
- A description of any restrictions on data availability
- For clinical datasets or third party data, please ensure that the statement adheres to our policy

All data and additional information required to reanalyze the data reported in this paper are available upon request. Further information and requests for resources and reagents should be directed to and will be fulfilled by the corresponding author Samuel K. McBrayer (samuel.mcbrayer@utsouthwestern.edu).

Plasmids generated in this study are available at Addgene.

Metabolomics data are publicly available at the National Metabolomics Data Repository (NMDR), the Metabolomics Workbench, under project accession number PR002945. RNA sequencing data are publicly available at the Gene Expression Omnibus (GEO) under accession number GSE320388. Proteomics data are publicly available at the Mass Spectrometry Interactive Virtual Environment (MassIVE) under accession number MSV000100431.

Research involving human participants, their data, or biological material

Policy information about studies with human participants or human data. See also policy information about sex, gender (identity/presentation), and sexual orientation and race, ethnicity and racism.

Reporting on sex and gender	Sex of human participants in explant stable isotope tracing studies are reported in Supplementary Table 4.
Reporting on race, ethnicity, or other socially relevant groupings	Race, ethnicity, and other socially relevant groupings are not reported for explant stable isotope tracing studies.
Population characteristics	Age, brain regions, and molecular features of human samples in explant stable isotope tracing studies are reported in Supplementary Table 4.
Recruitment	Participants in explant stable isotope tracing experiments were recruited from patients undergoing neurosurgical procedures at the University of Pittsburgh Medical Center.
Ethics oversight	Patient tissue and blood were collected following ethical and technical guidelines on the use of human samples for biomedical research at the University of Pittsburgh Medical Center after informed patient consent under a protocol approved by the University of Pittsburgh Medical Center's Institutional Review Board.

Note that full information on the approval of the study protocol must also be provided in the manuscript.

Field-specific reporting

Please select the one below that is the best fit for your research. If you are not sure, read the appropriate sections before making your selection.

☒ Life sciences ☐ Behavioural & social sciences ☐ Ecological, evolutionary & environmental sciences

For a reference copy of the document with all sections, see nature.com/documents/nr-reporting-summary-flat.pdf

Life sciences study design

All studies must disclose on these points even when the disclosure is negative.

Sample size	Sample size was determined based on availability of samples and a minimum of three biological replicates to ensure sufficient statistical power.
Data exclusions	No data were excluded.
Replication	Multiple biologic replicates were performed for each experiment (as noted in each figure legend).
Randomization	All human explant tissues, cell cultures, and mice were assigned to experimental groups randomly.
Blinding	Investigators were not blinded during sample collection or analysis. Sample processing was equivalent for all samples.

Reporting for specific materials, systems and methods

We require information from authors about some types of materials, experimental systems and methods used in many studies. Here, indicate whether each material, system or method listed is relevant to your study. If you are not sure if a list item applies to your research, read the appropriate section before selecting a response.

Materials & experimental systems

n/a	Involved in the study
☐	☒ Antibodies
☐	☒ Eukaryotic cell lines
☒	☐ Palaeontology and archaeology
☐	☒ Animals and other organisms
☒	☐ Clinical data
☒	☐ Dual use research of concern
☒	☐ Plants

Methods

n/a	Involved in the study
☒	☐ ChIP-seq
☒	☐ Flow cytometry
☒	☐ MRI-based neuroimaging

Antibodies

Antibodies used

Primary antibodies used included: anti-IDH1 R132H (Dianova DIA-H09, 1:500, Mouse monoclonal, RRID: AB_2335716), anti-vinculin (Sigma V9131, 1:100,000, mouse monoclonal, RRID:AB_477629), anti-Phospho-CAD (Ser1859) (Cell Signaling Technologies 70307, 1:1,000, rabbit monoclonal, RRID: AB_2799782), anti-CAD (Cell Signaling Technologies 11933, 1:1,000, rabbit polyclonal, RRID: AB_2797772), anti-DHODH (Proteintech 14877, 1:1,000, rabbit polyclonal, RRID: AB_2091723), anti-UMPS (Millipore Sigma HPA036179, 1:1,000, rabbit polyclonal, RRID: AB_10673615), anti-UMPS (Proteintech 14830, 1:1,000, rabbit polyclonal, RRID: AB_2212392), anti-HA (Thermo Fisher 26183, 1:1,000, mouse monoclonal, RRID: AB_10978021), anti-HA (BioLegend 901513, 1:1,000, mouse monoclonal, RRID: AB_2565335), anti-FLAG (Millipore Sigma F1804, 1:1,000, mouse monoclonal, RRID: AB_262044), anti-Phospho-S6 ribosomal protein (Ser240/244) (Cell Signaling Technologies 5364, 1:1,000, rabbit monoclonal, RRID: AB_10694233), anti-S6 ribosomal protein (Cell Signaling Technologies 2217, 1:1,000, rabbit monoclonal, RRID: AB_331355), anti-Phospho-p70 S6 kinase (Thr389) (Cell Signaling Technologies 9234, 1:1,000, rabbit monoclonal, RRID: AB_2269803), and anti-p70 S6 kinase (Cell Signaling Technologies 2708, 1:1,000, rabbit monoclonal, RRID: AB_390722). HRP-conjugated secondary antibodies used included: anti-Mouse IgG (Thermo Fisher 31430, 1:2,000, goat polyclonal, RRID: AB_228307) and anti-Rabbit IgG (Thermo Fisher 31460, 1:2,000, goat polyclonal, RRID: AB_228341).

Validation

anti-IDH1 R132H antibody was validated in Figure 1c
anti-CAD, anti-DHODH, and anti-UMPS antibodies were validated in Extended Data Figure 7a
anti-Phospho-CAD (Ser1859), anti-Phospho-S6 ribosomal protein (Ser240/244), anti-S6 ribosomal protein, anti-Phospho-p70 S6 kinase (Thr389), and anti-p70 S6 kinase were validated in Extended Data Figure 7k
anti-vinculin, anti-FLAG, and anti-HA antibodies were validated by manufacturer by Western blot analysis

Eukaryotic cell lines

Policy information about cell lines and Sex and Gender in Research

Cell line source(s)

NHA Donor #1 cells (human astrocytes immortalized with HPV E6 and E7 and hTERT, sex unknown) were obtained from R. Pieper at the University of California San Francisco. NHA Donor #2 cells were generated from commercially obtained primary human astrocytes (Lonza CC-3187). HEK293T (female, ATCC CRL-3216, RRID: CVCL_0063), BJ (male, ATCC CRL-2522, RRID: CVCL_3653), HNF primary fibroblast (Lonza NHDF-Neo), and HeLa (female, Millipore Sigma 93021013, RRID: CVCL_0030) cells were obtained commercially. G9C CHO cells were a gift of R. Possemato at New York University. BT054 (female, RRID: CVCL_N707) cells were obtained from S. Weiss at the University of Calgary. HK157 (female) and HK308 (male) cells were obtained from H. Kornblum at the University of California Los Angeles. TS516 (sex unknown, RRID: CVCL_A5HY) and TS603 (sex unknown, RRID: CVCL_A5HW) cells were obtained from I. Mellinghoff at Memorial Sloan-Kettering Cancer Center. ENSA (sex unknown) and NSC11 (sex unknown) cells were obtained from J. Rich at University of North Carolina-Chapel Hill. MGG152 (male) cells were obtained from D. Cahill at Massachusetts General Hospital. Murine GSC lines PC948-1 and PIC144-1 were derived from autochthonous astrocytomas that formed in a modified version of a genetically engineered mouse model.

Authentication

Cell line authentication was not performed because reference short term tandem repeat profiles have not been established for these lines.

Mycoplasma contamination

All cell lines were routinely evaluated for mycoplasma contamination with the e-Myco Mycoplasma PCR Detection Kit (Bulldog Bio 2523348), e-Myco PLUS Mycoplasma PCR Detection Kit (Bulldog Bio 25233), or MycoAlert Mycoplasma Detection Kit (Lonza LT07-318) and confirmed to be negative.

Commonly misidentified lines
(See ICLAC register)

None.

Animals and other research organisms

Policy information about studies involving animals; ARRIVE guidelines recommended for reporting animal research, and Sex and Gender in Research

Laboratory animals

Animal welfare assessments were carried out daily during treatment periods. Animals were housed in a pathogen-free environment between 20-26°C and at 30-70% humidity, with a 12 hour:12 hour light:dark cycle. Fox Chase SCID (Charles River 236, RRID: IMSR_CRL:236) mice pre-catheterized in the jugular vein with a one-channel 25-gauge vascular access button (Instech Laboratories VABM1B/25) were obtained from Charles River Laboratories at 8-10 weeks of age. C57BL/6J (RRID: IMSR_JAX:000664) mice were

	obtained from the UT Southwestern Mouse Breeding Core or Jackson Laboratories. Mice were housed together (2-5 mice of the same sex per cage) and provided free access to chow diet (Teklad 2916) and water.
Wild animals	No wild animals were used.
Reporting on sex	Mice of both male and female sexes were used and proportions of mouse sexes used in infusion experiments are reported in the Methods.
Field-collected samples	No field-collected samples were used.
Ethics oversight	All care and treatment of experimental animals were carried out in strict accordance with Good Animal Practice as defined by the US Office of Laboratory Animal Welfare and approved by the UT Southwestern Medical Center (protocols 2017-101840, 2019-102795, and 2022-102897) Institutional Animal Care and Use Committee.

Note that full information on the approval of the study protocol must also be provided in the manuscript.

Plants

Seed stocks	*Report on the source of all seed stocks or other plant material used. If applicable, state the seed stock centre and catalogue number. If plant specimens were collected from the field, describe the collection location, date and sampling procedures.*
Novel plant genotypes	*Describe the methods by which all novel plant genotypes were produced. This includes those generated by transgenic approaches, gene editing, chemical/radiation-based mutagenesis and hybridization. For transgenic lines, describe the transformation method, the number of independent lines analyzed and the generation upon which experiments were performed. For gene-edited lines, describe the editor used, the endogenous sequence targeted for editing, the targeting guide RNA sequence (if applicable) and how the editor was applied.*
Authentication	*Describe any authentication procedures for each seed stock used or novel genotype generated. Describe any experiments used to assess the effect of a mutation and, where applicable, how potential secondary effects (e.g. second site T-DNA insertions, mosiacism, off-target gene editing) were examined.*
