# Supplementary material for: Nitrogen metabolism profiling reveals cell state-specific pyrimidine synthesis pathway choice
Source: Nat Metab. 2026 Apr 29;8(5):1124–48. doi: 10.1038/s42255-026-01520-0 (PMC13218935; doi:10.1038/s42255-026-01520-0)

Figure 1c:
IDH1-R132H portion of blot
Molecular weight marker

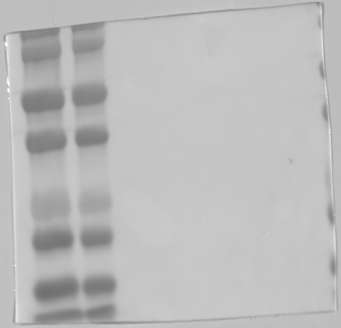

Figure 1c:
IDH1-R132H portion of blot
Chemiluminescence

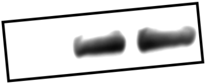

Figure 1c:
vinculin portion of blot
Molecular weight marker

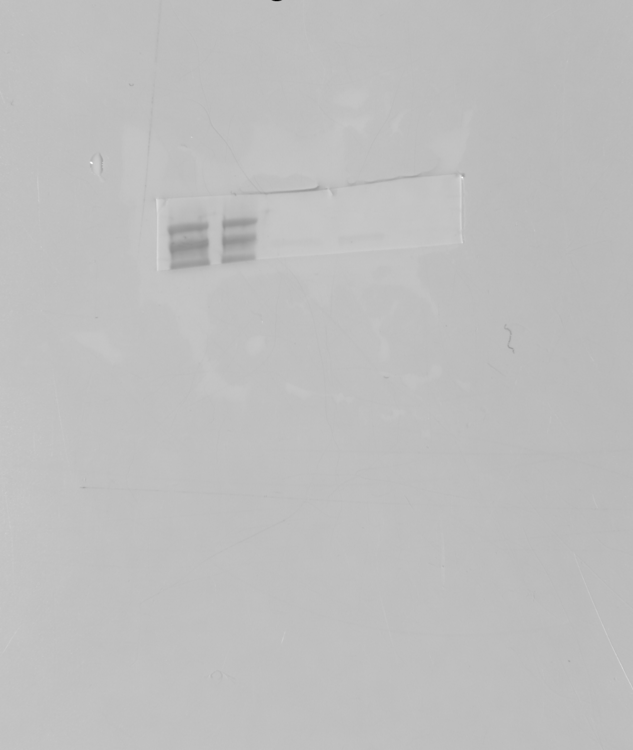

Figure 1c:
vinculin portion of blot
Chemiluminescence

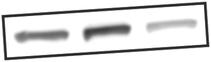

Supplement: Supplementary file 12 — Unprocessed western blots. [file 42255_2026_1520_MOESM12_ESM.pdf]
