# Supplementary material for: Nitrogen metabolism profiling reveals cell state-specific pyrimidine synthesis pathway choice
Source: Nat Metab. 2026 Apr 29;8(5):1124–48. doi: 10.1038/s42255-026-01520-0 (PMC13218935; doi:10.1038/s42255-026-01520-0)

Figure 5p:
CAD/vinculin portion of blot
Molecular weight marker

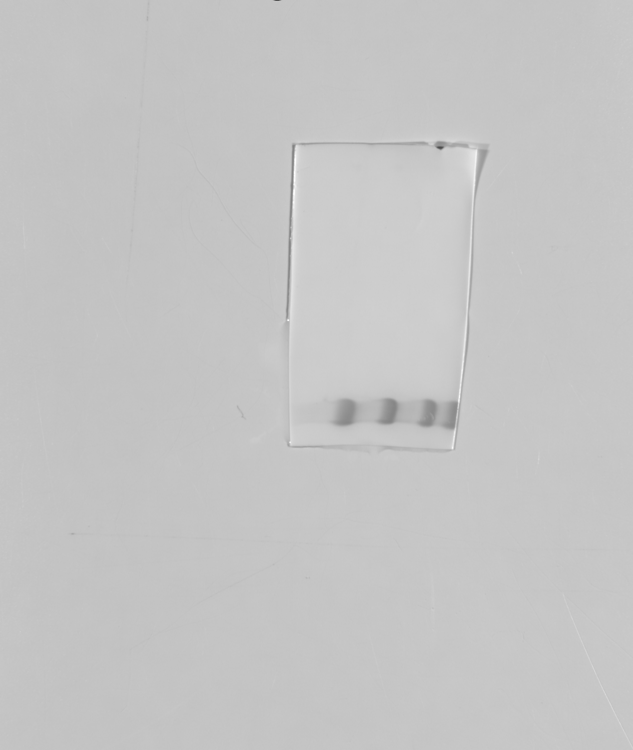

Figure 5p:
CAD/vinculin portion of blot
Chemiluminescence – CAD

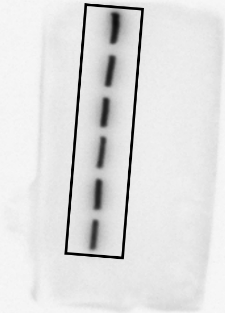

Figure 5p:
DHODH/UMPS portion of blot
Molecular weight marker

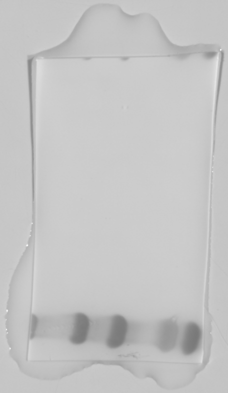

Figure 5p:
DHODH/UMPS portion of blot
Chemiluminescence – DHODH

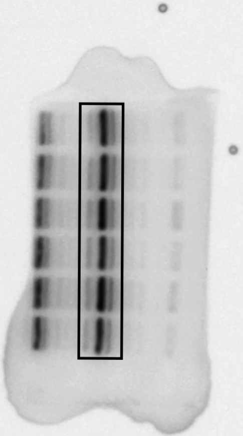

Figure 5p:
DHODH/UMPS portion of blot
Molecular weight marker

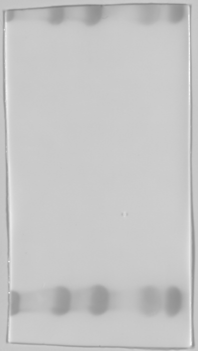

Figure 5p:
DHODH/UMPS portion of blot
Chemiluminescence – UMPS

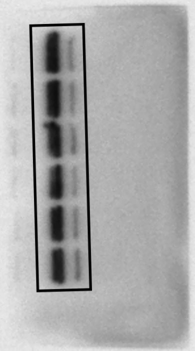

Figure 5p:
CAD/vinculin portion of blot
Molecular weight marker

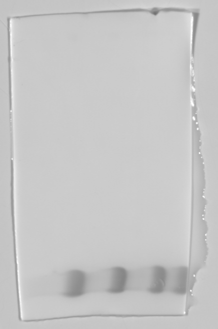

Figure 5p:
CAD/vinculin portion of blot
Chemiluminescence – vinculin

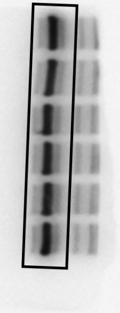

Supplement: Supplementary file 17 — Unprocessed western blots. [file 42255_2026_1520_MOESM17_ESM.pdf]
