# Supplementary material for: Nitrogen metabolism profiling reveals cell state-specific pyrimidine synthesis pathway choice
Source: Nat Metab. 2026 Apr 29;8(5):1124–48. doi: 10.1038/s42255-026-01520-0 (PMC13218935; doi:10.1038/s42255-026-01520-0)

Figure 6d:
CAD/FLAG portion of blot
Molecular weight marker

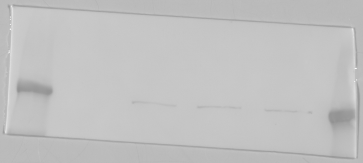

Figure 6d:
CAD/FLAG portion of blot
Chemiluminescence – FLAG

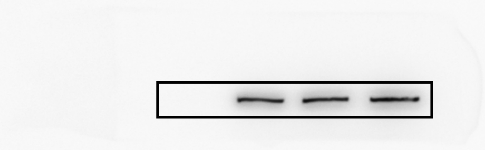

Figure 6d:
CAD/FLAG portion of blot
Chemiluminescence – CAD

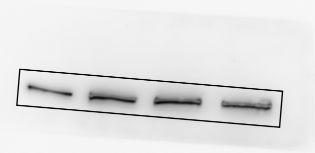

Figure 6d:
Vinculin portion of blot
Molecular weight marker

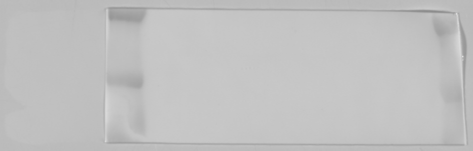

Figure 6d:
Vinculin portion of blot
Chemiluminescence – vinculin

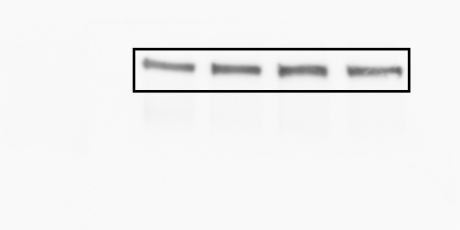

Supplement: Supplementary file 19 — Unprocessed western blots. [file 42255_2026_1520_MOESM19_ESM.pdf]
