# Supplementary material for: Nitrogen metabolism profiling reveals cell state-specific pyrimidine synthesis pathway choice
Source: Nat Metab. 2026 Apr 29;8(5):1124–48. doi: 10.1038/s42255-026-01520-0 (PMC13218935; doi:10.1038/s42255-026-01520-0)

Extended Data Figure 7a:
CAD/vinculin portion of blot
Molecular weight marker

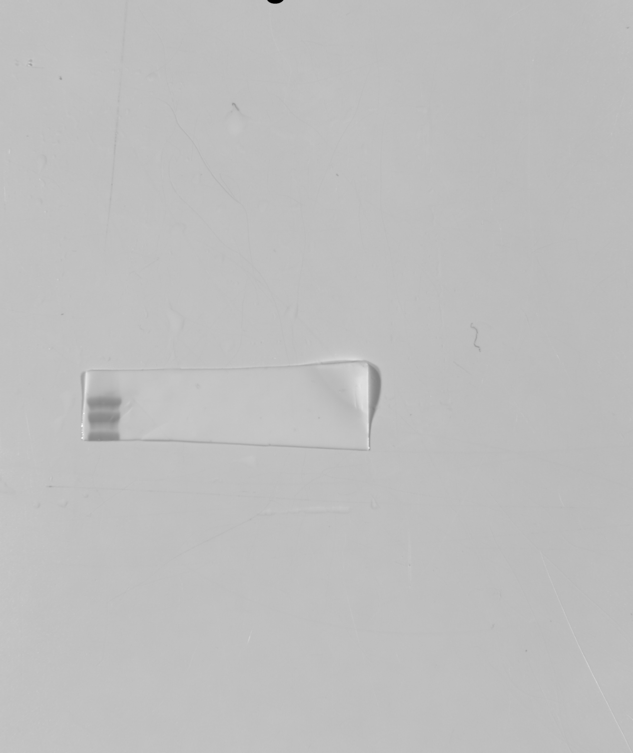

Extended Data Figure 7a:
CAD/vinculin portion of blot
Chemiluminescence – CAD

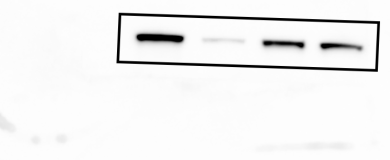

Extended Data Figure 7a:
DHODH/UMPS portion of blot
Molecular weight marker

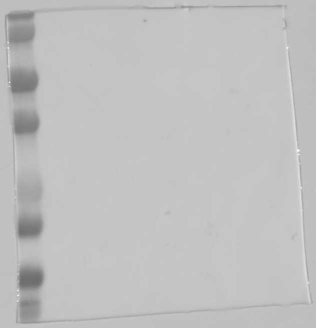

Extended Data Figure 7a:
DHODH/UMPS portion of blot
Chemiluminescence – DHODH

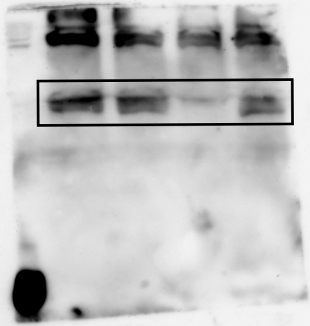

Extended Data Figure 7a:
DHODH/UMPS portion of blot
Molecular weight marker

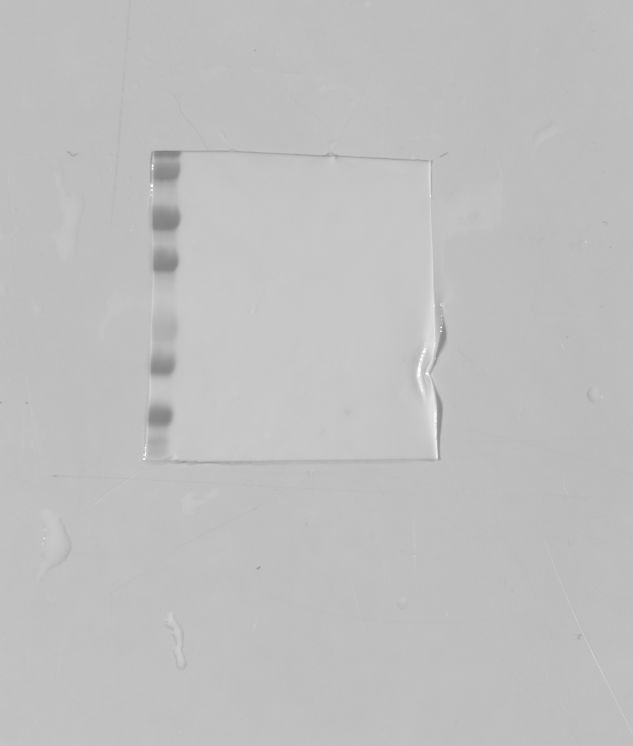

Extended Data Figure 7a:
DHODH/UMPS portion of blot
Chemiluminescence – UMPS

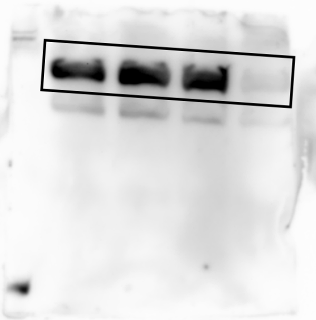

Extended Data Figure 7a:
CAD/vinculin portion of blot
Molecular weight marker

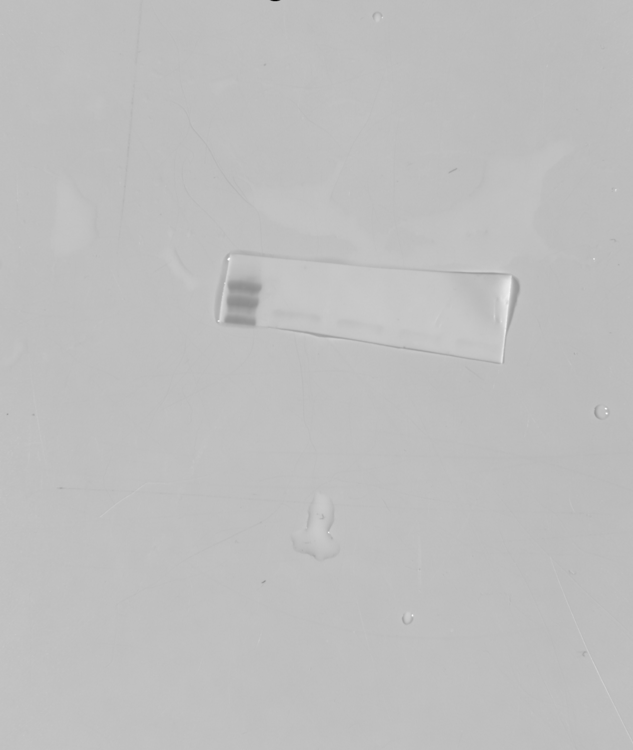

Extended Data Figure 7a:
CAD/vinculin portion of blot
Chemiluminescence – vinculin

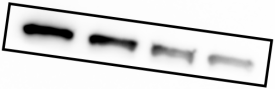

Extended Data Figure 7b:
CAD/vinculin portion of blot
Molecular weight marker

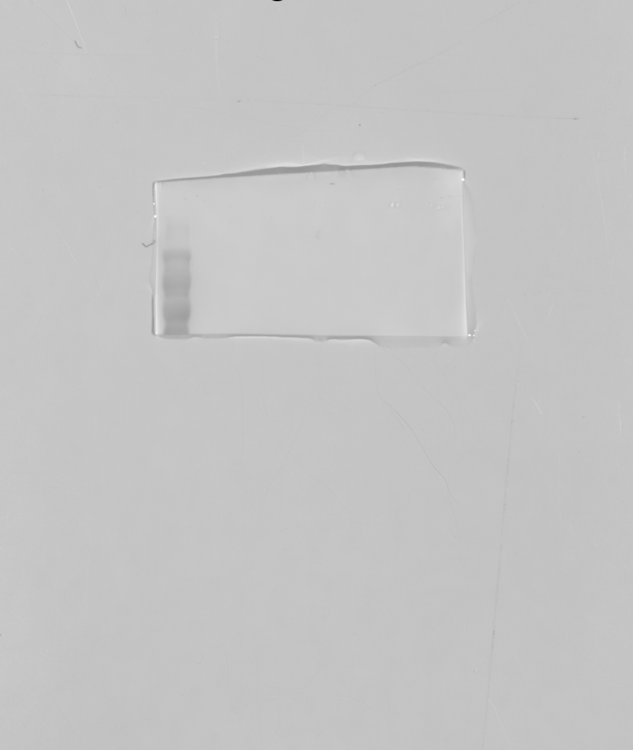

Extended Data Figure 7b:
CAD/vinculin portion of blot
Chemiluminescence – CAD

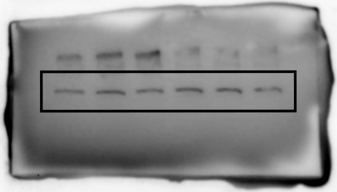

Extended Data Figure 7b:
DHODH/UMPS portion of blot
Molecular weight marker

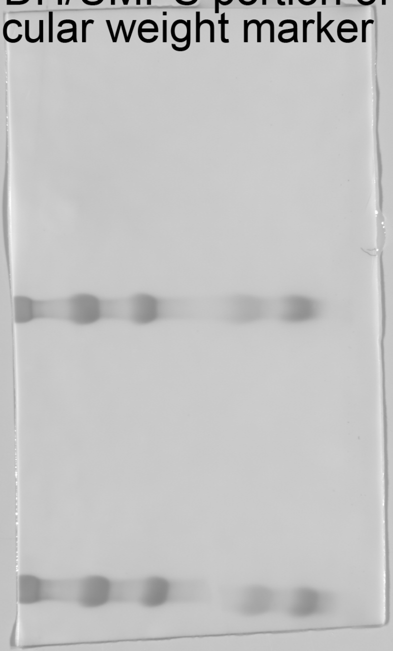

Extended Data Figure 7b:
DHODH/UMPS portion of blot
Chemiluminescence – DHODH

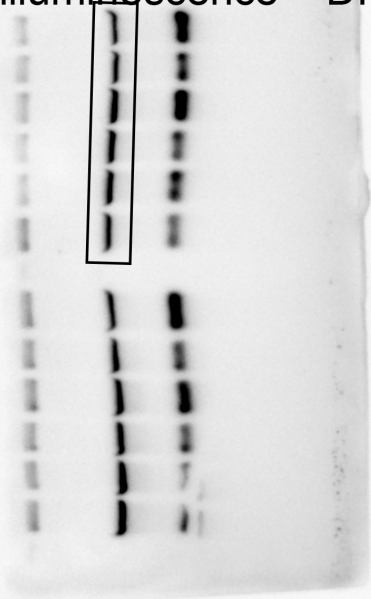

Extended Data Figure 7b:
DHODH/UMPS portion of blot
Molecular weight marker

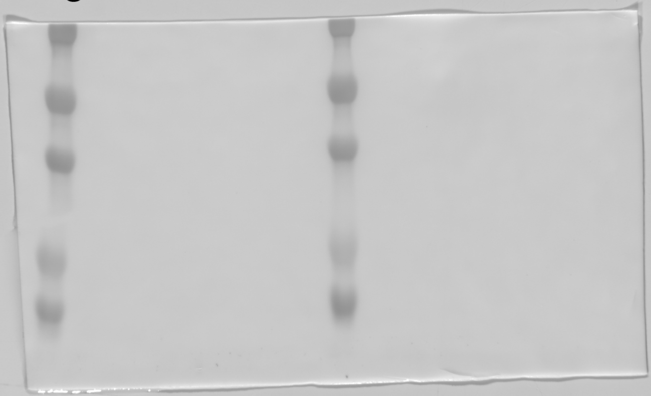

Extended Data Figure 7b:
DHODH/UMPS portion of blot
Chemiluminescence – UMPS

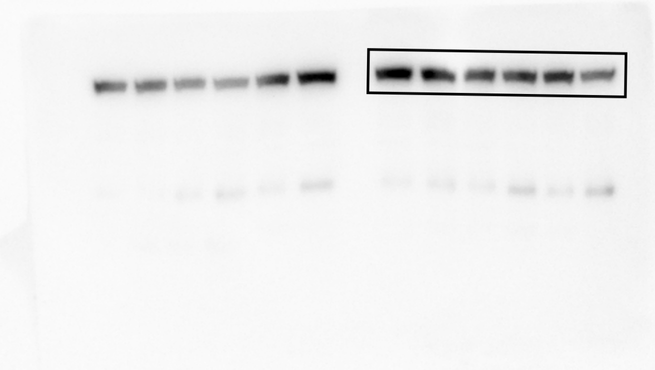

Extended Data Figure 7b:
CAD/vinculin portion of blot
Molecular weight marker

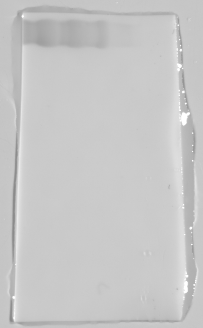

Extended Data Figure 7b:
CAD/vinculin portion of blot
Chemiluminescence – vinculin

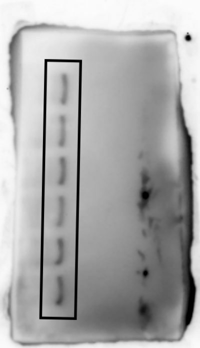

Extended Data Figure 7c:
CAD/vinculin portion of blot
Molecular weight marker

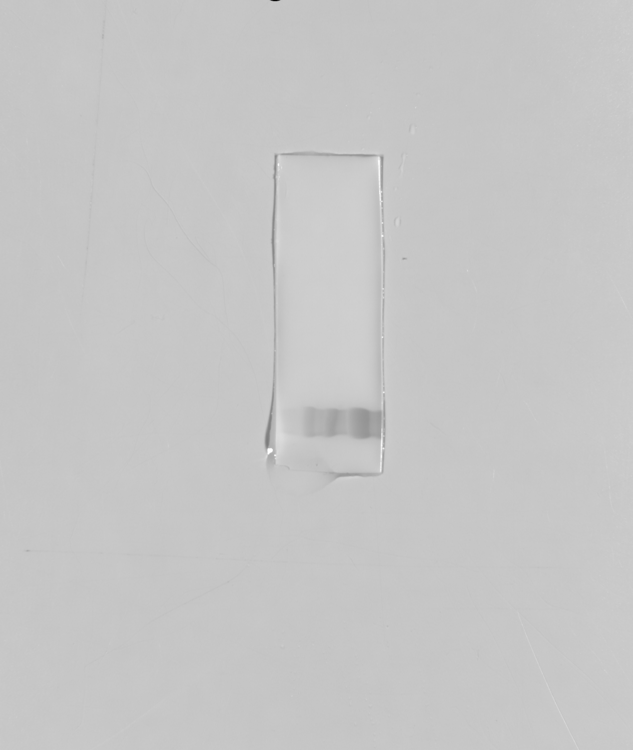

Extended Data Figure 7c:
CAD/vinculin portion of blot
Chemiluminescence – CAD

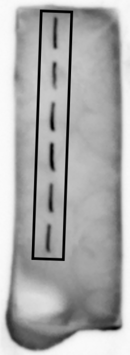

Extended Data Figure 7c:
DHODH/UMPS portion of blot
Molecular weight marker

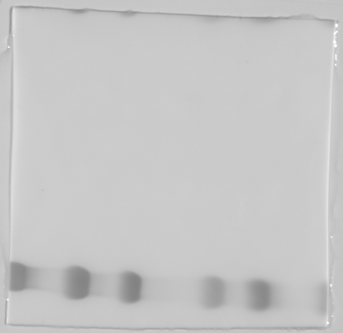

Extended Data Figure 7c:
DHODH/UMPS portion of blot
Chemiluminescence – DHODH

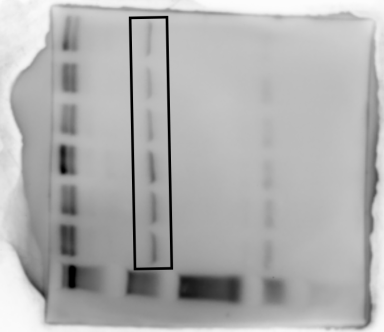

Extended Data Figure 7c:
DHODH/UMPS portion of blot
Molecular weight marker

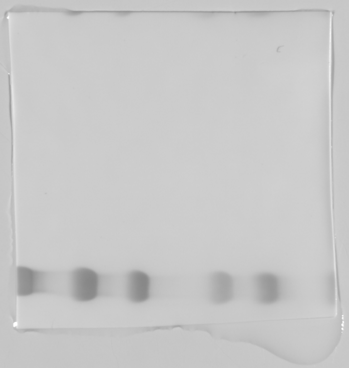

Extended Data Figure 7c:
DHODH/UMPS portion of blot
Chemiluminescence – UMPS

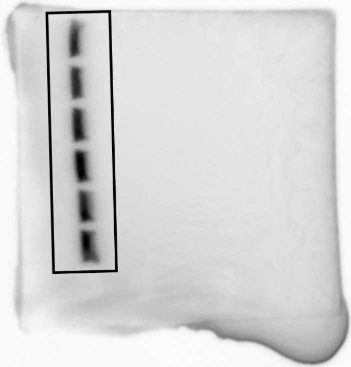

Extended Data Figure 7c:
CAD/vinculin portion of blot
Molecular weight marker

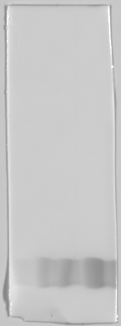

Extended Data Figure 7c:
CAD/vinculin portion of blot
Chemiluminescence – vinculin

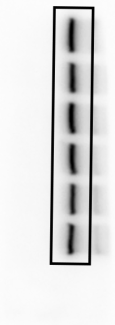

Extended Data Figure 7d:
CAD/vinculin portion of blot
Molecular weight marker

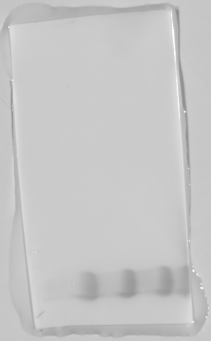

Extended Data Figure 7d:
CAD/vinculin portion of blot
Chemiluminescence – CAD

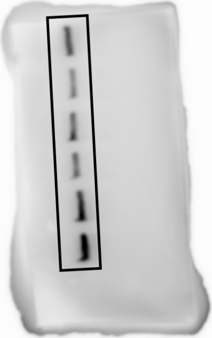

Extended Data Figure 7d:
DHODH/UMPS portion of blot
Molecular weight marker

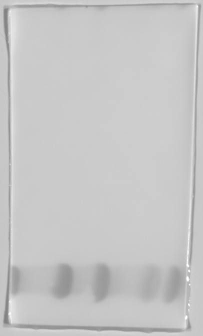

Extended Data Figure 7d:
DHODH/UMPS portion of blot
Chemiluminescence – DHODH

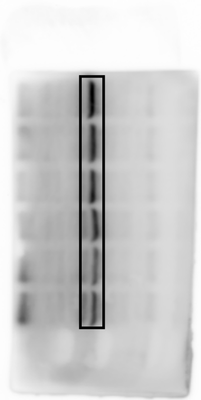

Extended Data Figure 7d:
DHODH/UMPS portion of blot
Molecular weight marker

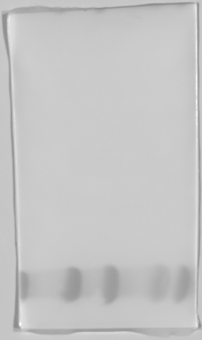

Extended Data Figure 7d:
DHODH/UMPS portion of blot
Chemiluminescence – UMPS

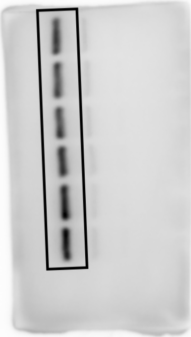

Extended Data Figure 7d:
CAD/vinculin portion of blot
Molecular weight marker

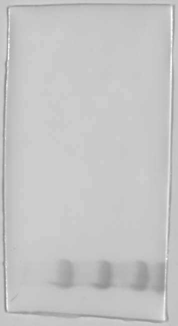

Extended Data Figure 7d:
CAD/vinculin portion of blot
Chemiluminescence – vinculin

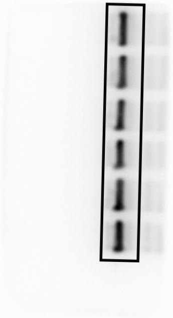

Extended Data Figure 7k:
CAD/vinculin portion of blot
Molecular weight marker

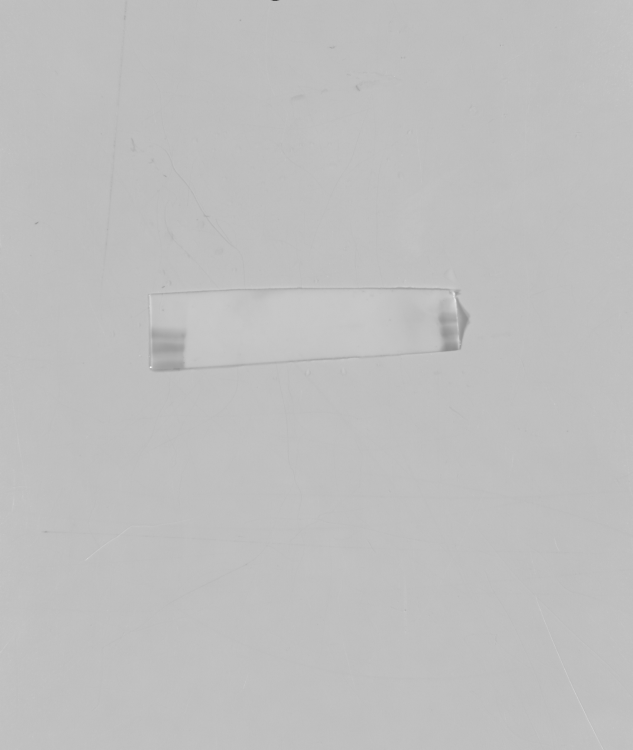

Extended Data Figure 7k:
CAD/vinculin portion of blot
Chemiluminescence – CAD pS1859

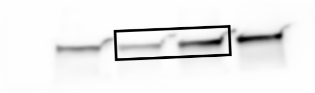

Extended Data Figure 7k:
CAD/vinculin portion of blot
Molecular weight marker

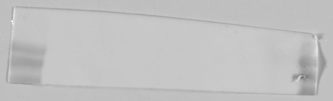

Extended Data Figure 7k:
CAD/vinculin portion of blot
Chemiluminescence – CAD

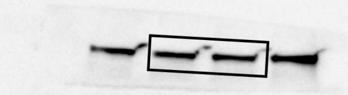

Extended Data Figure 7k:
S6 portion of blot
Molecular weight marker

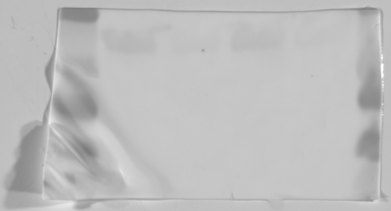

Extended Data Figure 7k:
S6 portion of blot
Chemiluminescence – S6 pS240

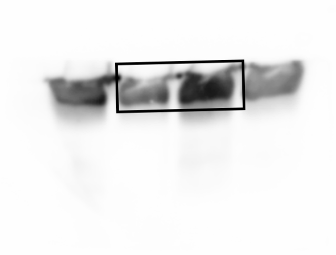

Extended Data Figure 7k:
S6 portion of blot
Molecular weight marker

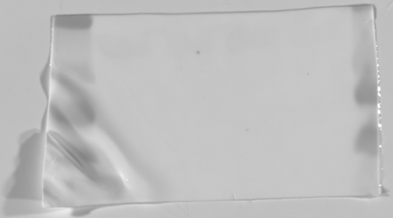

Extended Data Figure 7k:
S6 portion of blot
Chemiluminescence – S6

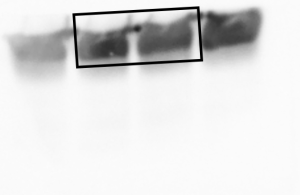

Extended Data Figure 7k:
S6K portion of blot
Molecular weight marker

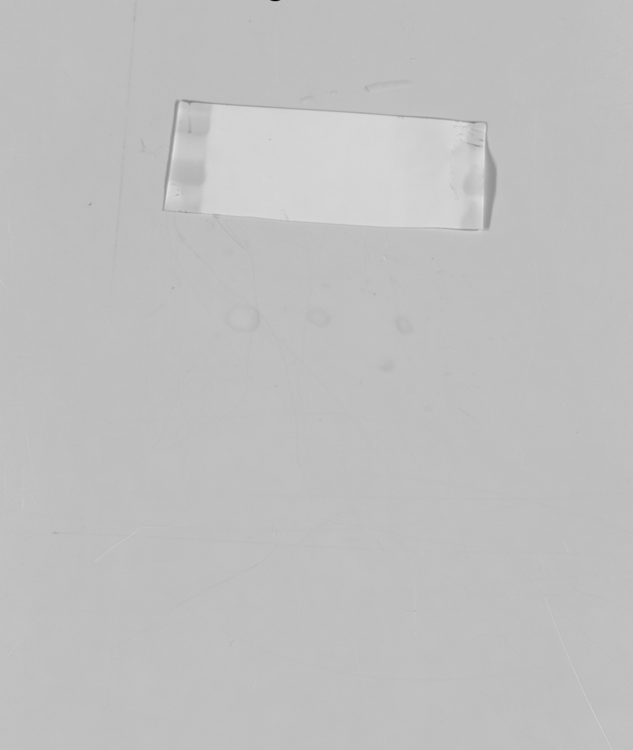

Extended Data Figure 7k:
S6K portion of blot
Chemiluminescence – S6K pT389

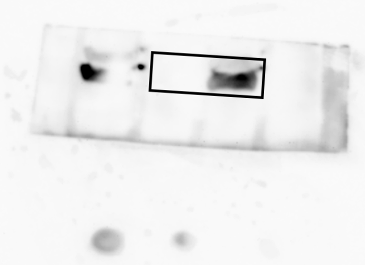

Extended Data Figure 7k:
S6K portion of blot
Molecular weight marker

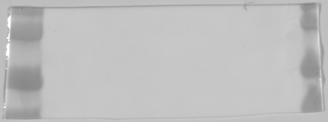

Extended Data Figure 7k:
S6K portion of blot
Chemiluminescence – S6K

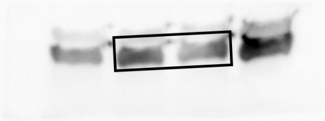

Extended Data Figure 7k:
CAD/vinculin portion of blot
Molecular weight marker

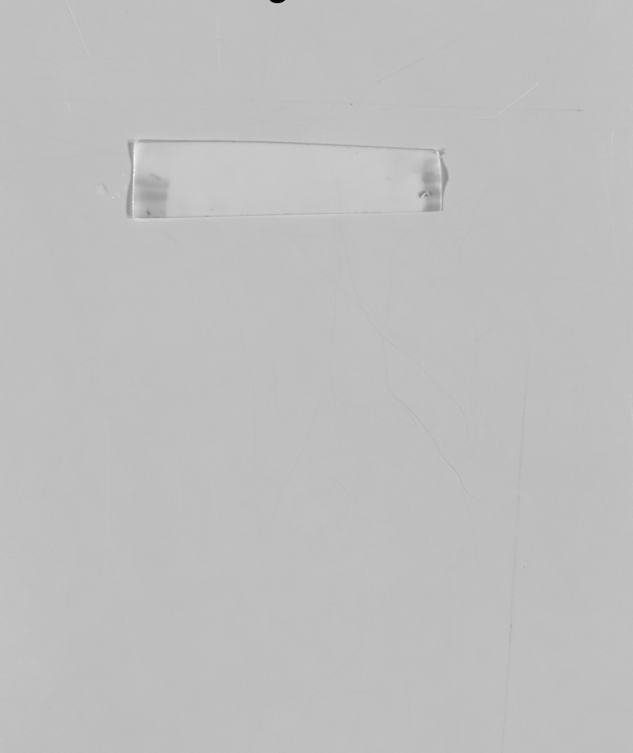

Extended Data Figure 7k:
CAD/vinculin portion of blot
Chemiluminescence – vinculin

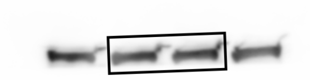

Extended Data Figure 7l:
S6 portion of blot
Molecular weight marker

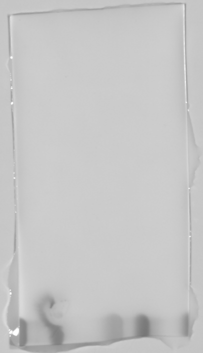

Extended Data Figure 7l:
S6 portion of blot
Chemiluminescence – S6 pS240

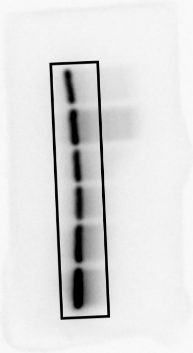

Extended Data Figure 7l:
S6 portion of blot
Molecular weight marker

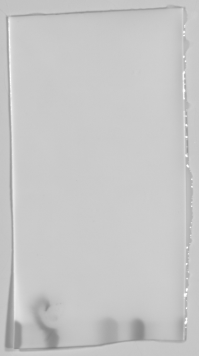

Extended Data Figure 7l:
S6 portion of blot
Chemiluminescence – S6

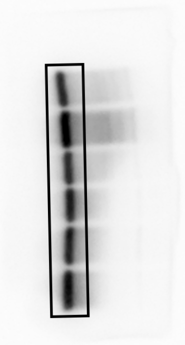

Extended Data Figure 7l:
S6K/vinculin portion of blot
Molecular weight marker

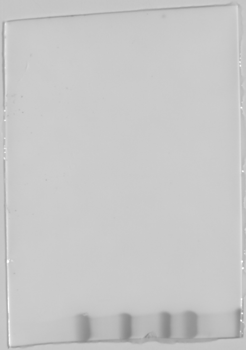

Extended Data Figure 7l:
S6K/vinculin portion of blot
Chemiluminescence – S6K pT389

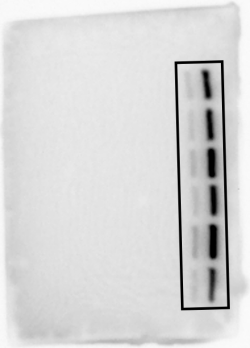

Extended Data Figure 7l:
S6K/vinculin portion of blot
Molecular weight marker

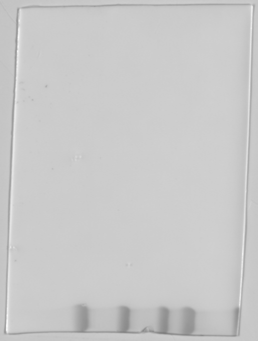

Extended Data Figure 7l:
S6K/vinculin portion of blot
Chemiluminescence – S6K

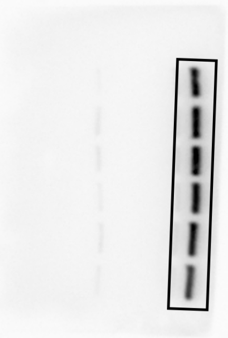

Extended Data Figure 7l:
S6K/vinculin portion of blot
Molecular weight marker

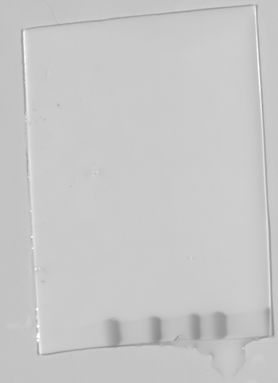

Extended Data Figure 7l:
S6K/vinculin portion of blot
Chemiluminescence – vinculin

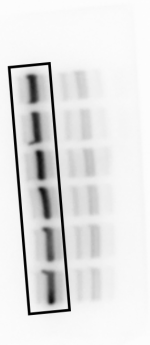

Extended Data Figure 7m:
S6 portion of blot
Molecular weight marker

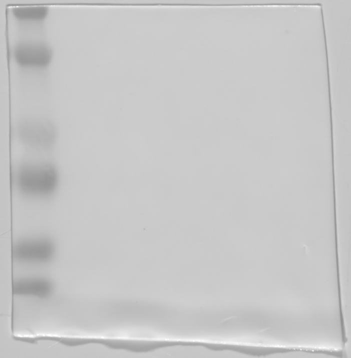

Extended Data Figure 7m:
S6 portion of blot
Chemiluminescence – S6 pS240

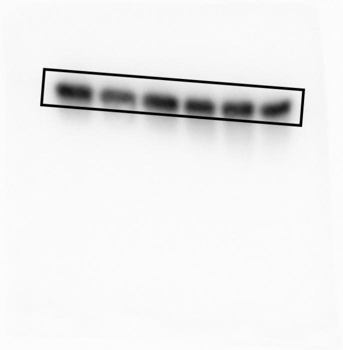

Extended Data Figure 7m:
S6 portion of blot
Molecular weight marker

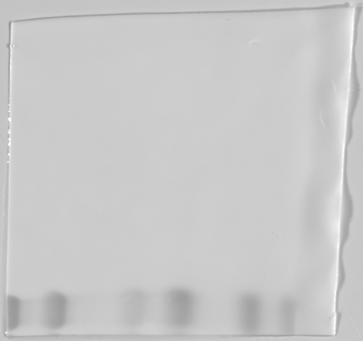

Extended Data Figure 7m:
S6 portion of blot
Chemiluminescence – S6

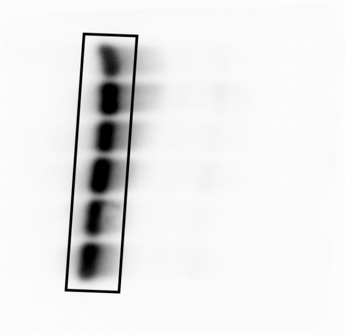

Extended Data Figure 7m:
S6K/vinculin portion of blot
Molecular weight marker

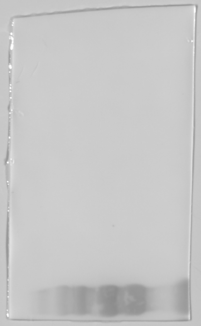

Extended Data Figure 7m:
S6K/vinculin portion of blot
Chemiluminescence – S6K
pT389

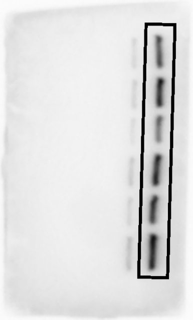

Extended Data Figure 7m:
S6K/vinculin portion of blot
Molecular weight marker

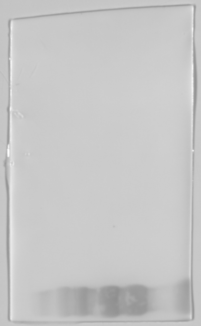

Extended Data Figure 7m:
S6K/vinculin portion of blot
Chemiluminescence – S6K

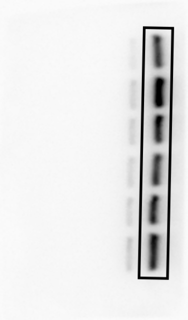

Extended Data Figure 7m:
S6K/vinculin portion of blot
Molecular weight marker

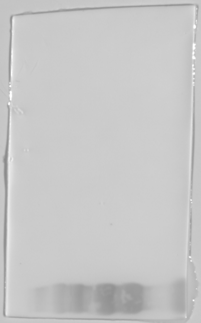

Extended Data Figure 7m:
S6K/vinculin portion of blot
Chemiluminescence – vinculin

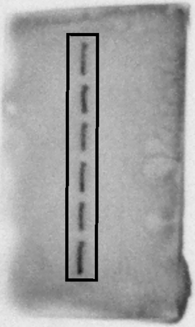

Extended Data Figure 7n:
S6 portion of blot
Molecular weight marker

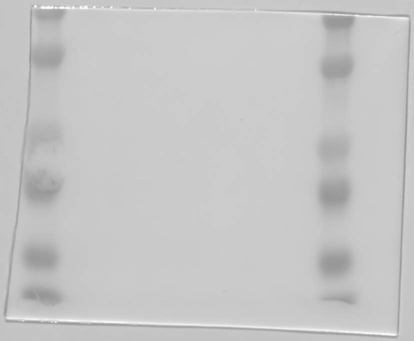

Extended Data Figure 7n:
S6 portion of blot
Chemiluminescence – S6 pS240

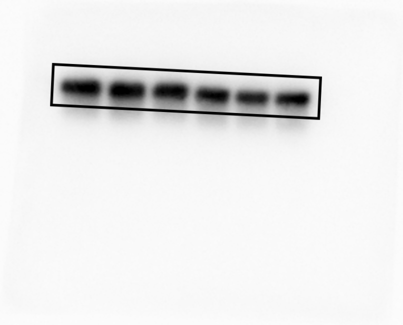

Extended Data Figure 7n:
S6 portion of blot
Molecular weight marker

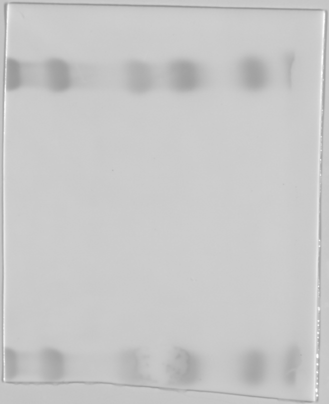

Extended Data Figure 7n:
S6 portion of blot
Chemiluminescence – S6

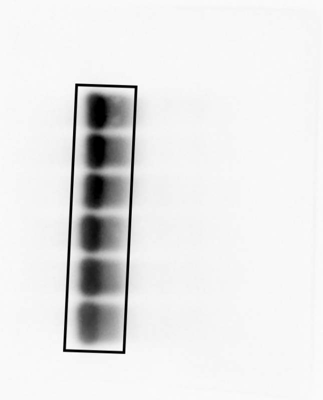

Extended Data Figure 7n:
S6K/vinculin portion of blot
Molecular weight marker

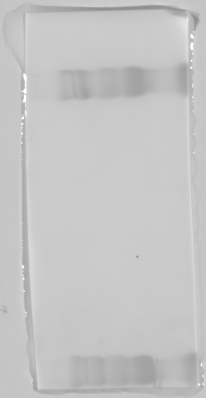

Extended Data Figure 7n:
S6K/vinculin portion of blot
Chemiluminescence – S6K
pT389

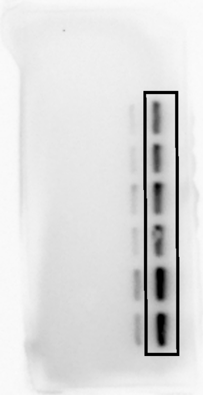

Extended Data Figure 7n:
S6K/vinculin portion of blot
Molecular weight marker

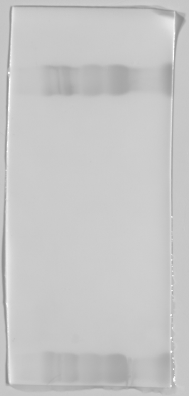

Extended Data Figure 7n:
S6K/vinculin portion of blot
Chemiluminescence – S6K

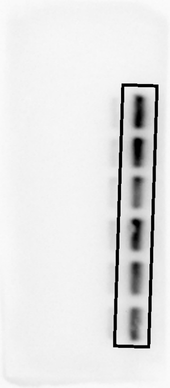

Extended Data Figure 7n:
S6K/vinculin portion of blot
Molecular weight marker

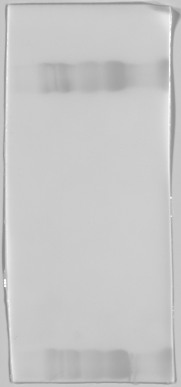

Extended Data Figure 7n:
S6K/vinculin portion of blot
Chemiluminescence – vinculin

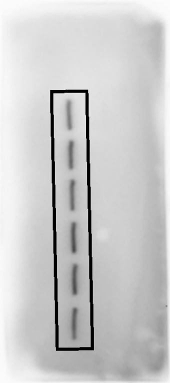

Extended Data Figure 7o:
S6 portion of blot
Molecular weight marker

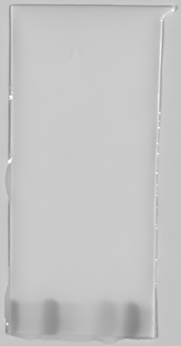

Extended Data Figure 7o:
S6 portion of blot
Chemiluminescence – S6 pS240

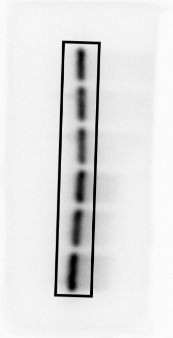

Extended Data Figure 7o:
S6 portion of blot
Molecular weight marker

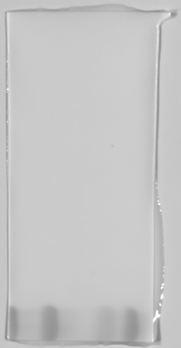

Extended Data Figure 7o:
S6 portion of blot
Chemiluminescence – S6

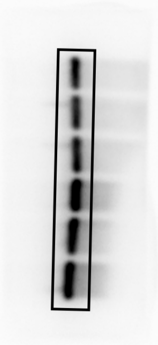

Extended Data Figure 7o:
S6K/vinculin portion of blot
Molecular weight marker

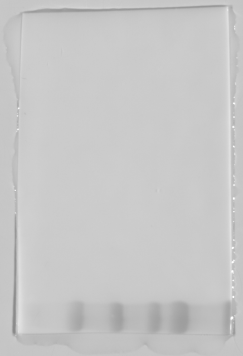

Extended Data Figure 7o:
S6K/vinculin portion of blot
Chemiluminescence – S6K pT389

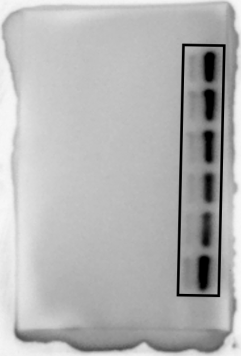

Extended Data Figure 7o:
S6K/vinculin portion of blot
Molecular weight marker

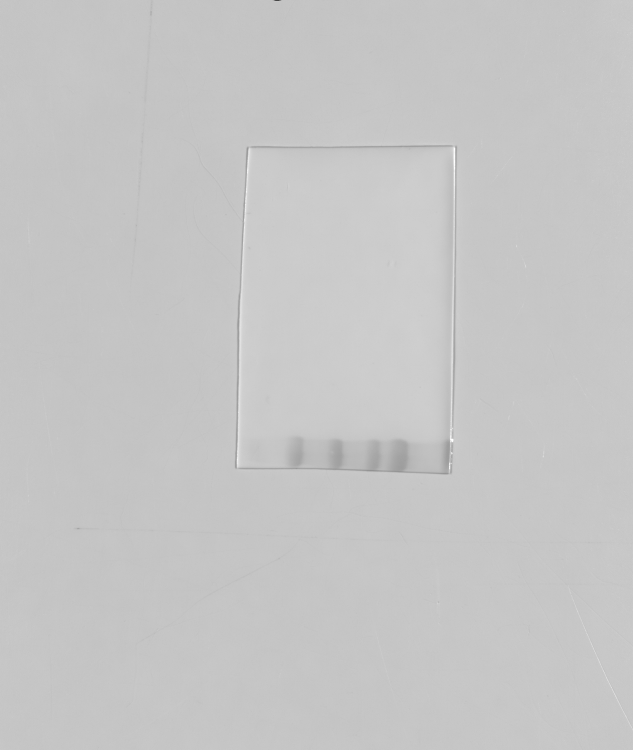

Extended Data Figure 7o:
S6K/vinculin portion of blot
Chemiluminescence – S6K

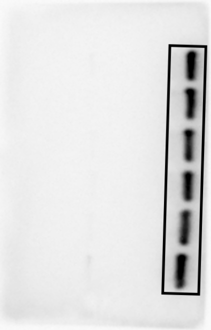

Extended Data Figure 7o:
S6K/vinculin portion of blot
Molecular weight marker

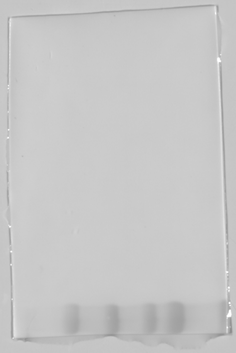

Extended Data Figure 7o:
S6K/vinculin portion of blot
Chemiluminescence – vinculin

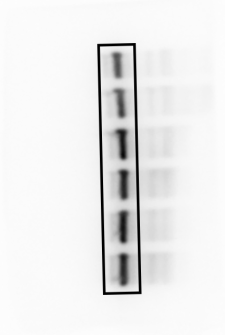

Extended Data Figure 7p:
Molecular weight marker

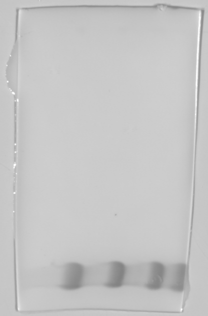

Extended Data Figure 7p:
Chemiluminescence – CAD
pS1859

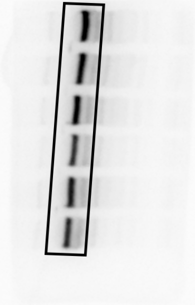

Extended Data Figure 7p:
Molecular weight marker

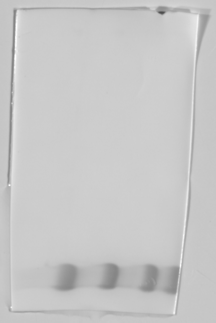

Extended Data Figure 7p:
Chemiluminescence – CAD

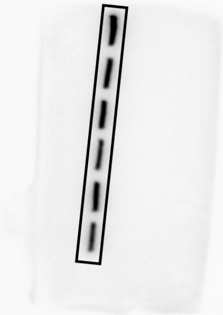

Extended Data Figure 7p:
Molecular weight marker

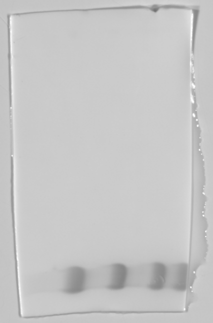

Extended Data Figure 7p:
Chemiluminescence – vinculin

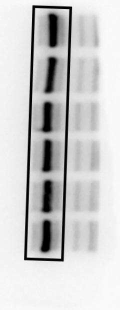

Extended Data Figure 7q:
Molecular weight marker

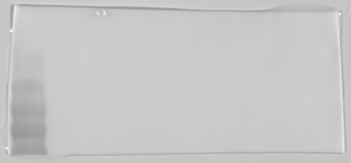

Extended Data Figure 7q:
Chemiluminescence – CAD

pS1859

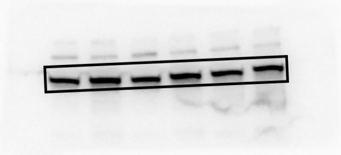

Extended Data Figure 7q:
Molecular weight marker

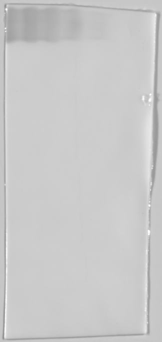

Extended Data Figure 7q:
Chemiluminescence – CAD

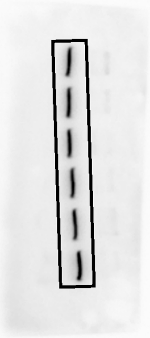

Extended Data Figure 7q: Molecular weight marker

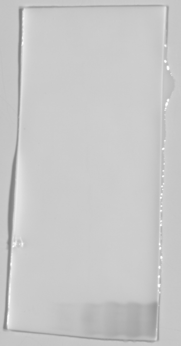

Extended Data Figure 7q:
Chemiluminescence – vinculin

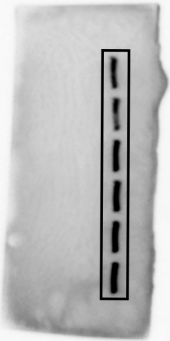

Extended Data Figure 7r:
Molecular weight marker

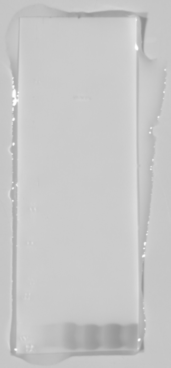

Extended Data Figure 7r:
Chemiluminescence – CAD

pS1859

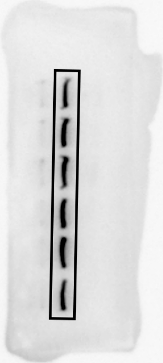

Extended Data Figure 7r:
Molecular weight marker

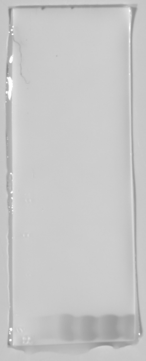

Extended Data Figure 7r: Chemiluminescence – CAD

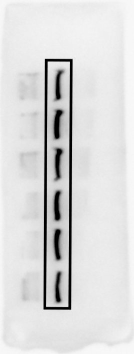

Extended Data Figure 7r:
Molecular weight marker

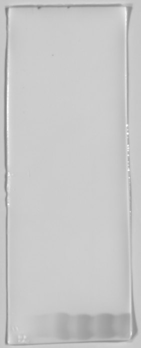

Extended Data Figure 7r:
Chemiluminescence – vinculin

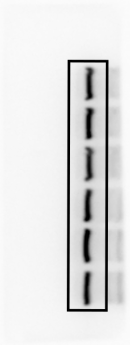

Extended Data Figure 7s: Molecular weight marker

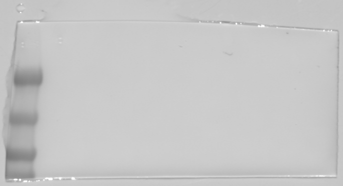

Extended Data Figure 7s: Chemiluminescence – CAD pS1859

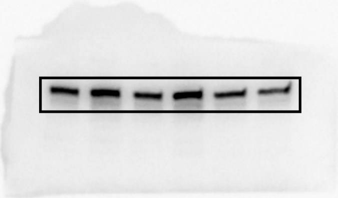

Extended Data Figure 7s: Molecular weight marker

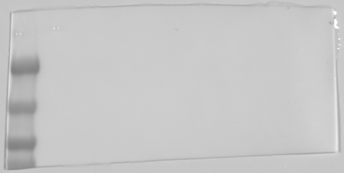

Extended Data Figure 7s: Chemiluminescence – CAD

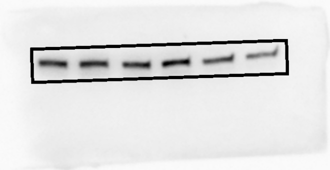

Extended Data Figure 7s: Molecular weight marker

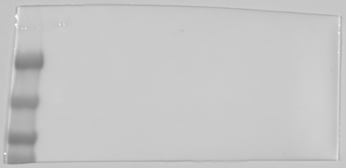

Extended Data Figure 7s: Chemiluminescence – vinculin

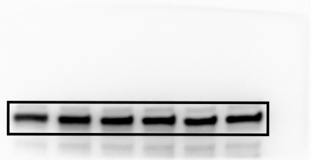

Supplement: Supplementary file 28 — Unprocessed western blots. [file 42255_2026_1520_MOESM28_ESM.pdf]
