# Supplementary material for: Nitrogen metabolism profiling reveals cell state-specific pyrimidine synthesis pathway choice
Source: Nat Metab. 2026 Apr 29;8(5):1124–48. doi: 10.1038/s42255-026-01520-0 (PMC13218935; doi:10.1038/s42255-026-01520-0)

Extended Data Figure 8a:
Molecular weight marker

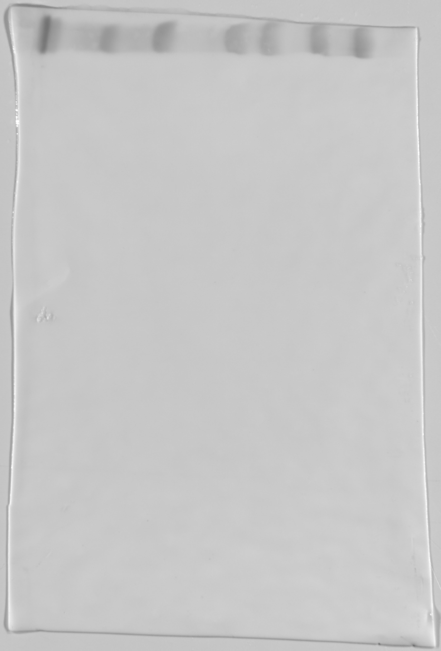

Extended Data Figure 8a: Chemiluminescence – CAD

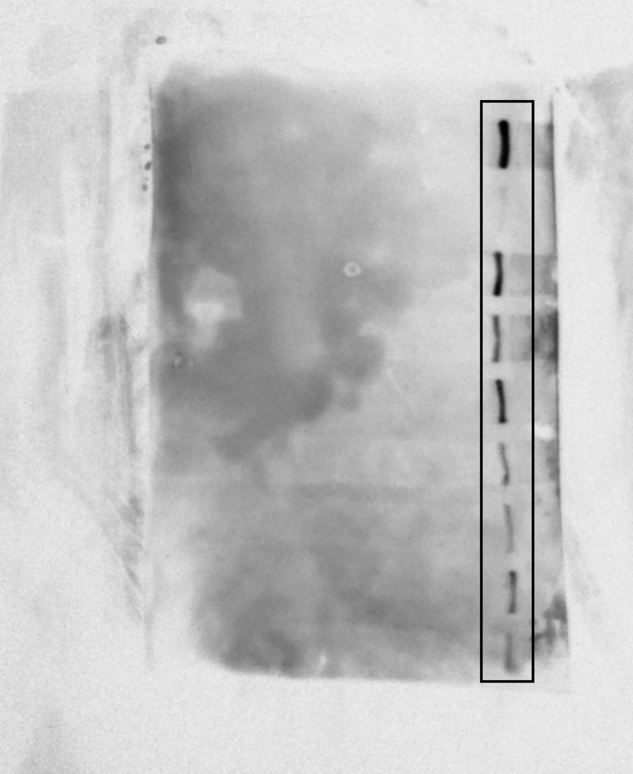

Extended Data Figure 8a:
Molecular weight marker

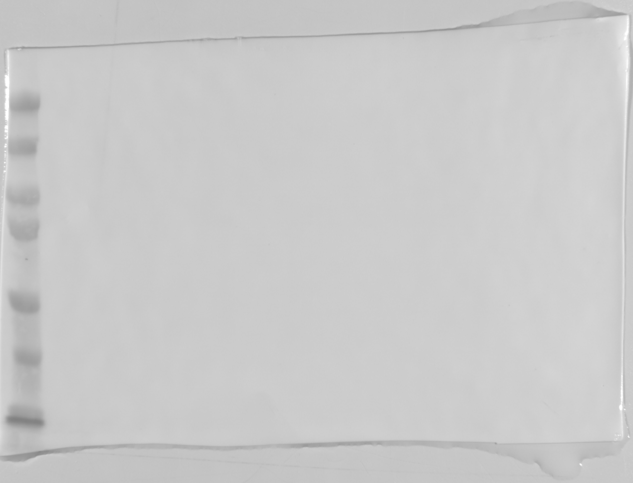

Extended Data Figure 8a:
Chemiluminescence – HA

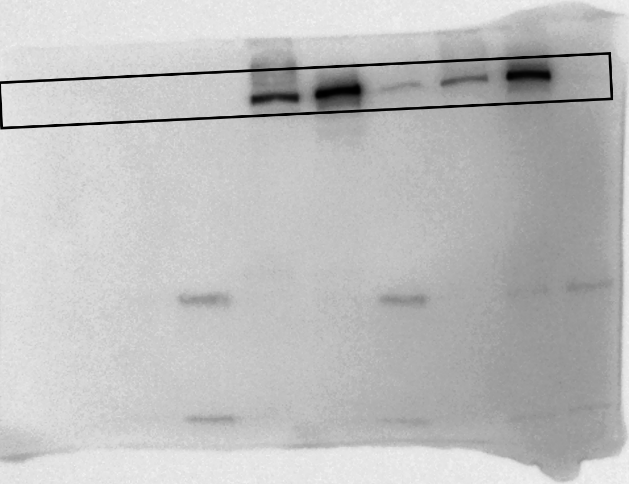

Extended Data Figure 8a:
Molecular weight marker

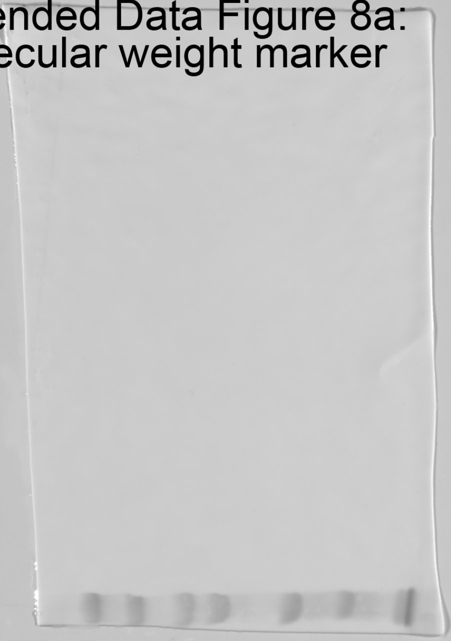

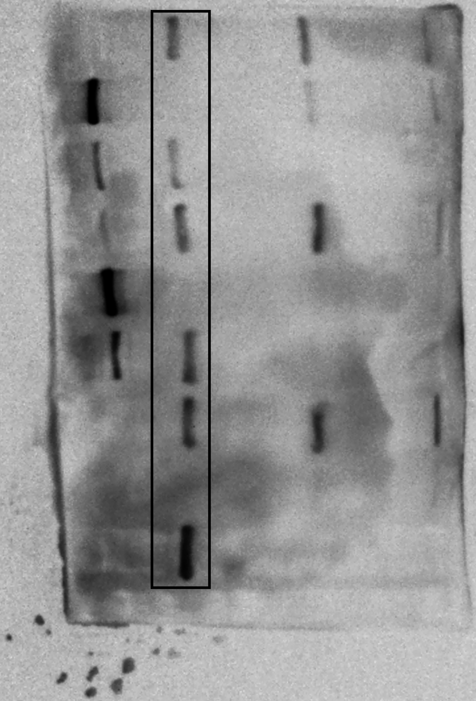

Extended Data Figure 8a:
Chemiluminescence – vinculin

Extended Data Figure 8e:
HA portion of blot
Molecular weight marker

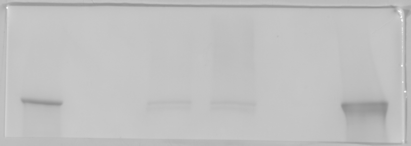

Extended Data Figure 8e:
HA portion of blot
Chemiluminescence – HA

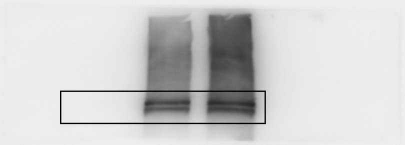

Extended Data Figure 8e:
Vinculin portion of blot
Molecular weight marker

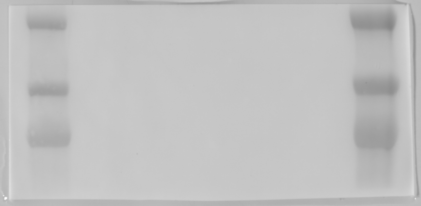

Extended Data Figure 8e:
Vinculin portion of blot
Chemiluminescence – vinculin

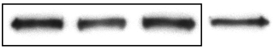

Supplement: Supplementary file 30 — Unprocessed western blots. [file 42255_2026_1520_MOESM30_ESM.pdf]
